# Possible Beneficial Effects of Hydrolyzable Tannins Deriving from *Castanea sativa* L. in Internal Medicine

**DOI:** 10.3390/nu16010045

**Published:** 2023-12-22

**Authors:** Giulia Marrone, Manuela Di Lauro, Francesco Izzo, Kevin Cornali, Claudia Masci, Chiara Vita, Francesco Occhiuto, Nicola Di Daniele, Antonino De Lorenzo, Annalisa Noce

**Affiliations:** 1 Department of Systems Medicine, University of Rome Tor Vergata, 00133 Rome, Italy; giulia.marrone@uniroma2.it (G.M.); manuela.dilauro@ptvonline.it (M.D.L.); fraizzo29@gmail.com (F.I.); cornali.kevin@hotmail.it (K.C.); masciclaudia@gmail.com (C.M.); didaniele@med.uniroma2.it (N.D.D.); 2 QuMAP (Quality of Goods and Product Reliability), University of Florence, PIN, 59100 Prato, Italy; chiara.vita@pin.unifi.it; 3 Department of Economics, Management and Business Law, University of Bari “Aldo Moro”, Piazza Umberto I, 70121 Bari, Italy; 4 Ph.D. School of Applied Medical-Surgical Sciences, University of Rome Tor Vergata, Via Montpellier 1, 00133 Rome, Italy; 5 Fondazione Leonardo per le Scienze Mediche Onlus, Policlinico Abano, 35031 Abano Terme, Italy; 6 Section of Clinical Nutrition and Nutrigenomic, Department of Biomedicine and Prevention, University of Rome Tor Vergata, 00133 Rome, Italy; 7 UOSD Nephrology and Dialysis, Policlinico Tor Vergata, 00133 Rome, Italy

**Keywords:** chestnuts, hydrolyzable tannins, antioxidant effects, anticancer effects, cardioprotective effects, nutraceuticals, lipid metabolism

## Abstract

Hydrolyzable tannins (HTs) deriving from chestnuts have demonstrated, through numerous studies, the ability to exert multiple beneficial effects, including antioxidant and antimicrobial effects, on the lipid metabolism and cancer cells. The latter effect is very fascinating, since different polyphenols deriving from chestnuts were able to synergistically induce the inhibition of cancerous cells through multiple pathways. Moreover, the main mechanisms by which tannins induce antioxidant functions include: the reduction in oxidative stress, the ability to scavenge free radicals, and the modulation of specific enzymes, such as superoxide dismutase. HTs have also been shown to exert significant antimicrobial activity by suppressing microbial growth. The actions on the lipid metabolism are several, among which is the inhibition of lipid accumulation. Thus, tannins seem to induce a cardioprotective effect. In fact, through various mechanisms, such as the relaxation of the vascular smooth muscle, HTs were proven to be efficient against arterial hypertension. Therefore, the great number of studies in this field prove the growing interest on the utilization of natural bioactive compounds, such as HTs deriving from natural sources or obtained by circular economy models, as potential nutraceuticals or adjuvants therapies.

## 1. Introduction

Tannins are water-soluble polyphenols commonly found within many sources in nature. These compounds include oligomers or polymers of flavanol and phenolic acid monomers, and have different sensory/chemical properties and functions compared to their monomers. Tannins are encountered as plant secondary metabolites and can be divided into two main categories: condensed and hydrolyzable [1]. The distinction between the two groups relies on chemical aspects, such as the resistance to hydrolysis and the chemical stability [2,3].

**Figure 1:**
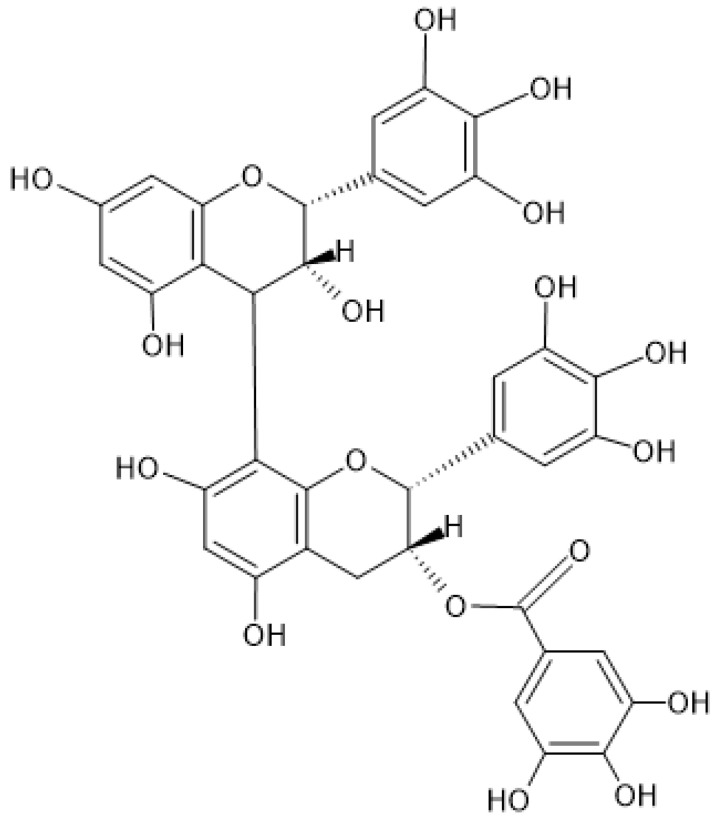
Chemical structure of prodelphinidin B-2 3′-O-gallate.

Tannin condensates are oligomers (from 2 to 10 monomers) or polymers (>10 monomers) of flavan-3-ol units that, based on the positioning of their -OH and -H groups, can give rise to procyanidins, prodelphinidins, prorobinetinidins, and prodelfinidins (Figure 1). For instance, the procyanidins contain catechin or epicatechin, while the prodelfinidins contain gallocatechin or epigallocatechin [2].

**Figure 2:**
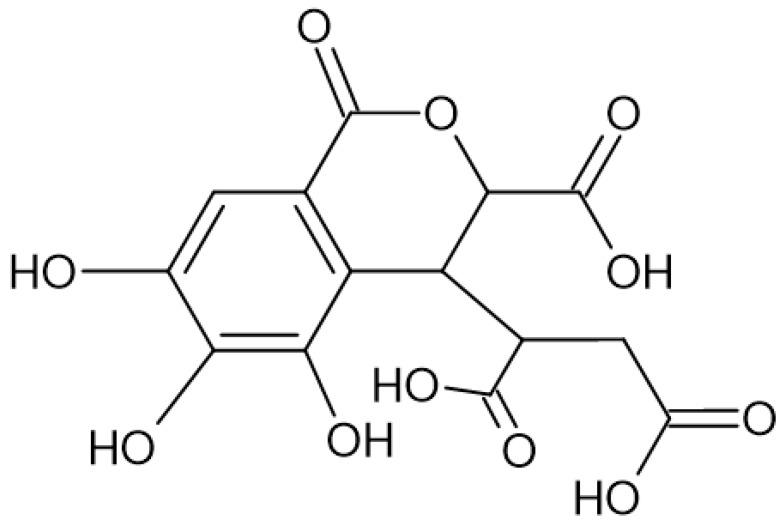
Chemical structure of chebulic acid, which pertains to the group of ellagitannins.

**Figure 3:**
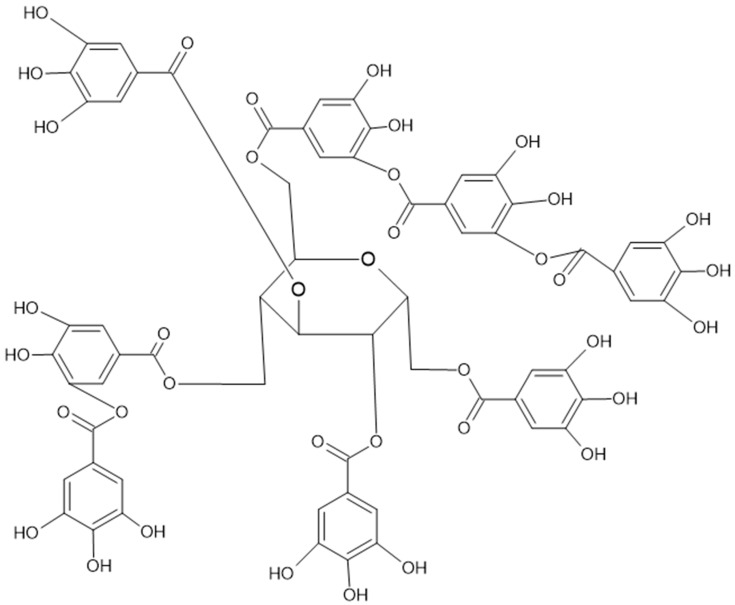
Complex gallotannin, named tannic acid, also known as Chinese tannin, is highly present in chestnuts.

The hydrolyzable tannins (HTs) are hydrolyzed by acids into glucose and ellagic acid or into glucose and gallic acid (GA). The HT group includes ellagitannins (ETs) (Figure 2) and gallotannins (Figure 3).

The HTs deriving from *Castanea sativa* L. have shown to induce multiple beneficial effects on both human and animal health [4]. The genus *Castanea* consists of twelve species of deciduous trees, all native of the temperate regions of the northern hemisphere. Among them, *Castanea sativa*, also known as European or sweet chestnut, is the most emblematic species present in the southern area of Europe. Italy is the country which produces the highest quantity of chestnuts [5]. 

The fruit of the chestnut is enclosed in a thorny shell and is composed of many elements, among which are the leathery pericarp and two thin integuments covering two large fleshy cotyledons. All the components of the chestnut, including the waste and byproducts, are rich in bioactive compounds, with several beneficial properties. For this reason, the chestnut represents an ideal candidate in various fields, like in the pharmaceutical and cosmetic industries [5].

**Figure 4:**
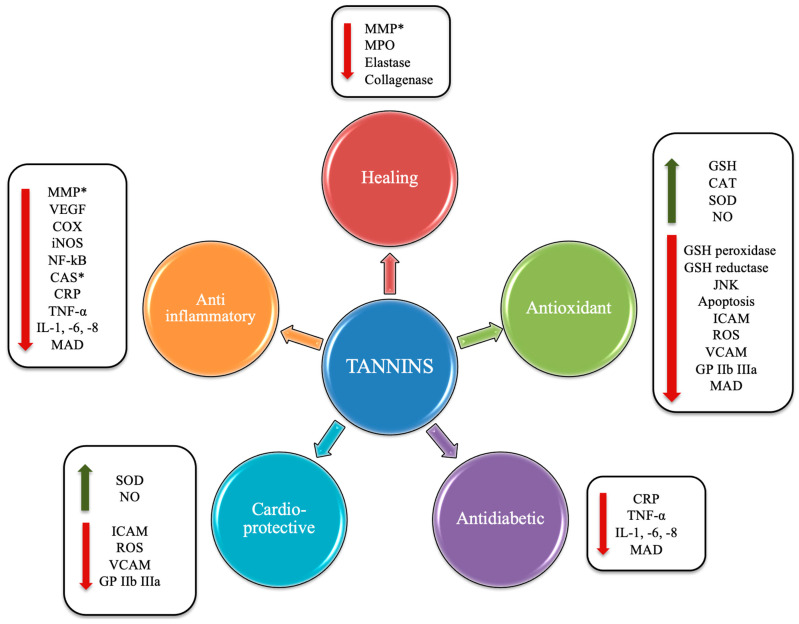
Potential beneficial effects induced by tannins. Abbreviations: *, antioxidant capacity; VEGF, vascular endothelial growth factor; MMP, matrix metalloproteinase; COX, cyclooxygenase; iNOS, inducible nitric oxide synthase; NF-κB, nuclear factor kappa-light-chain-enhancer of activated B cells; CAS, caspase; CRP, C-reactive protein; TNF-α, tumor necrosis factor-α; IL, interleukin; MAD, malondialdehyde; GP IIb IIIa, glycoprotein IIb/IIIa; NO, nitric oxide; VCAM, vascular cell adhesion protein; ROS, reactive oxygen species; ICAM, intercellular adhesion molecule; SOD, superoxide dismutase; CAT, catalase; JNK, C-Jun N-terminal kinase.

The health effects of these compounds (Figure 4) lie in the fact that the chestnut and its derivatives are rich in HTs. The latter have been shown to exert antioxidant and antimicrobial actions, antineoplastic effects, to favor the lipid metabolism [6], and to exert cardioprotective actions [7]. 

Clinical results obtained from human studies are just limited to some plant extracts. However, promising results about different biological effects have been shown through both in vitro and animal studies [6]. It is important to distinguish between high- and low-molecular-weight tannins. Those which have a lower molecular weight are more easily absorbable and they can induce systemic effects in different organs [6], whilst those which are nonabsorbable may induce beneficial effects only in the gastrointestinal tract [6]. 

Several studies have highlighted how gallic acid and ellagic acid, being the main compounds identified within chestnut extracts, were able, through different mechanisms, to induce antioxidant effects both in in vitro and in vivo studies [8,9,10,11,12]. Free-radical scavenging activity and hydrogen donation are the two known mechanisms able to inhibit lipid oxidation and to provide antioxidant effects [13]. 

Among the beneficial effects exerted by HTs, there is also the antimicrobial one. This is an important aspect that can help to counteract the emerging problem of antibiotic resistance. 

**Figure 5:**
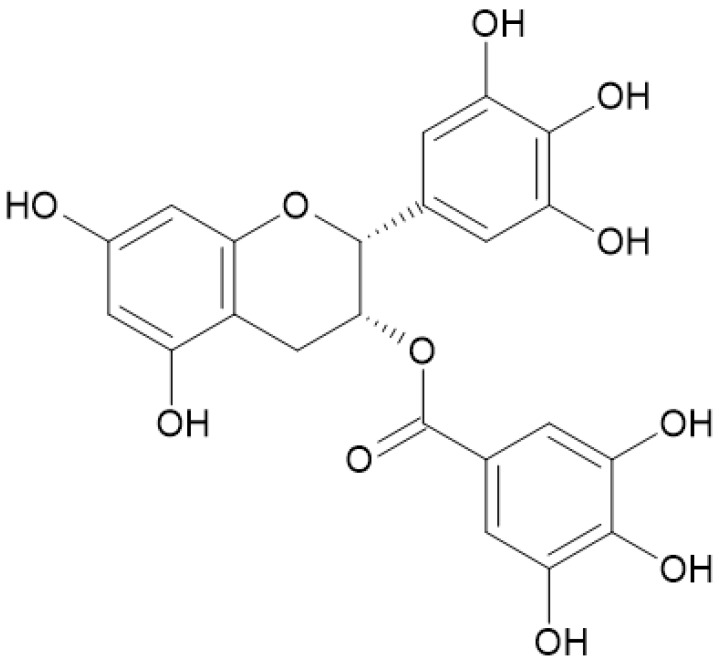
Epigallocatechin gallate is a catechin molecule with antioxidant and anti-inflammatory actions. It also protects the skin from UV-ray damage.

Moreover, it is very interesting how epigallocatechin gallate (Figure 5), a compound present within chestnuts, has shown to modulate chemotherapy-induced apoptosis in cancer of the bile duct [14]. 

Tannic acid has demonstrated the ability to synergistically provide anticancer effects in combination with some antineoplastic drugs. In detail, 5-fluorouracil and mytomycin-C, acting against human cholangiocarcinoma cells, modulate the drug efflux pathways, such as P-glycoprotein, which is one of the main mechanisms of neoplasia resistance [15]. 

Regarding the cardioprotective action, HTs have shown to induce vasorelaxation, as they are able to modulate the nitric oxide (NO) pathways [16,17]. In this context, HTs seem also to ameliorate the lipid metabolism. Rodrigues et al. [18] observed how the dietary supplementation of *Castanea sativa* was able to reduce abdominal adiposity in mice, improving their body composition. Yasuda et al. [19] observed how the administration of an extract consisting of water and chestnuts inhibited the accumulation of fat in 3T3-L1 cells and the lipase activity.

The aim of this review is to investigate the possible beneficial effects of HTs deriving from chestnuts obtained by circular economy models in internal medicine; in particular, our focus is centered on the positive actions in human health, such as the antioxidant, antimicrobial, and antineoplastic actions, and the effects on the lipid metabolism and cardioprotection.

## 2. Materials and Methods

We analyzed different sources of literature, focusing our interest on the relationship between keywords, such as “chestnut hydrolyzable tannins” [Title/Abstract] in combination with “internal medicine” AND “antioxidant effects” AND/OR “anti-microbial effects” AND/OR “lipid metabolism” AND/OR “cardio-protective effects” AND/OR “anti-neoplastic effects”, on the PubMed, Scopus, Cochrane Library, and Web of Science electronic databases.

The review and the reference lists were manually performed by the authors. The search was limited to English-language papers published up to August 2023.

## 3. The Impact of the Circular Economy on Tannin Production

The growing interest for sustainable policies has led to a higher implementation of circular economy models in different product sectors. Among these, the agroalimentary one plays an important role due to the elevated quantity of wastes and byproducts. 

Companies and industries have started to pursue specific objectives established by the United Nations 2030 Agenda, and for this reason, they follow these virtuous directives in conducting the different phases of the productive steps. This is aimed at generating the lowest possible environmental impact. The greater awareness of an ideal culture oriented towards environmental and economic sustainability represents a competitive and discriminating element that has brought many agricultural and agroalimentary companies to focus their attention on the multifunctional aspects of sustainability. 

Several scientific studies over the years have focused their attention on organic compounds deriving from the vegetal world generally used as waste or byproducts of the agricultural sector. Specifically, on polyphenols, which are secondary metabolites present within various tissues of all vegetal species [20].

The analytical characterization of waste products has allowed for the identification of numerous bioactive compounds present within single matrices. Different polyphenol compounds have been identified within vegetal species. Moreover, their biological properties and general characteristics have been discovered. These compounds, thanks to their properties, can be utilized as natural and biocompatible products [21]. This allows a minor usage of synthesized products whose safety is often questioned [22]. 

Chestnuts and the wood production chain give rise to matrices rich in tannins and their derivatives, which have shown multiple properties, such as antimicrobial, antioxidant, and astringent ones [23,24,25]. These properties, together with anti-inflammatory and antineoplastic ones [10,25,26], allow for the usage of such compounds in different sectors (textile/dyeing and furniture, agronomic, animal feeding, cosmetic, alimentary, and biomedical), both for traditional usages and for obtaining innovative and multifunctional products, where the chemical components, obtained from this synthesis, are either partially or totally substituted for natural molecules. The recycling of such bioactive components allows for the application of sustainability principles aimed not only at the recovery of waste products, but also at the implementation of solvent-free technologies. The extracts deriving from chestnut tannins (CTs) are obtained through an aqueous extraction method. This method permits the recovery the tannin fractions that permeate in the initial steps of the extraction process [27].

## 4. Tannins’ Mechanism of Action 

As previous described, HTs deriving from chestnuts have demonstrated the exertion of multiple beneficial effects both in human and animal studies.

The accumulation of reactive oxygen species (ROS) is commonly implicated with the occurrence of chronic degenerative non-communicable diseases, namely, pathological conditions related to systemic and chronic inflammation. Examples include: atherosclerosis, chronic kidney disease, cardiovascular disease, diabetes mellitus, and neurodegenerative pathologies, such as Parkinson’s and Alzheimer’s diseases [8]. 

The administration of natural antioxidants may help or serve as a supplement against oxidative mechanisms [8]. One possible mechanism, which induces antioxidant functions, is the ability to directly scavenge free radicals thanks to the donation of hydrogen atoms. Another possible antioxidant mechanism, induced by the administration of chestnut extracts, is the modulation of different enzymes normally involved in antioxidant functions. These enzymes include superoxide dismutase (SOD) and glutathione peroxidase (GSH-Px) [28]. The interaction between tannins and free radicals aims at creating resonance-stabilized phenix radicals, which confer important antioxidant properties. 

In recent years, the antimicrobial activity induced by some phenolic molecules has been a prominent element of research. This effect has been substantially associated to the hydroxyl groups present in these molecules. In fact, both the number and the position of hydroxyl groups present within phenolic molecules appear to be related to the level of their inhibitory capacity of bacterial growth [29]. The proposal of possible substitutes to combat bacterial strains resistant to conventional antibiotics has become fundamental [30]. Lima et al. [31] demonstrated how the interaction of phenolic compounds with host-gut microbiota results in a positive effect by increasing the beneficial bacterial population. According to Chanwitheesuk et al. [32], *Staphylococcus aureus* and *Salmonella* reported susceptibility to GA. Furthermore, the antimicrobial activity of phenolic molecules encountered within the chestnut extracts is exerted on different components of the bacterial cell, including some inactive essential enzymes and the cell membrane. These molecules are also able to modify specific functions of the genetic material of the bacteria. The Gram-negative bacterial cell wall is the principal obstacle for the entrance of the phenolic molecules into the cytoplasm because of the repulsion created between lipopolysaccharides and phenols [33].

Both hydrolyzable and condensed tannins have demonstrated the ability to inhibit the growth of several bacterial strains which are potentially pathogenic, such as *Escherichia coli*, without having any effects on the physiological growth of other gut beneficial bacteria [34]. 

Tannins have the ability to bind reversibly to some amino acids and dietary and endogenous proteins [35,36,37]. In this regard, a positive effect of both the HTs and the condensed ones on *Campylobacter jejuni* was demonstrated in vitro. Interestingly, it was also evaluated whether the amino acid supplementation would limit the tannin extracts’ activity. This experiment was performed to evaluate if the abundance of amino acids and soluble proteins within the gastrointestinal tract could interfere with the tannins’ antimicrobial action [38]. 

Moreover, polyphenolic molecules, including HTs, have also shown to exert antineoplastic properties. In fact, these molecules counteract different cancerous cell development [39], modulating several mechanisms, such as the regulation of cell signaling cascades. Among these are the transcription factors and various kinase enzymes that modulate the expression of genes involved in apoptosis. In addition, they are also able to check the interaction between growth factors and target receptors involved in specific signaling effects [39]. HTs used as antineoplastic agents have various advantages, including the specificity of the biological response and low toxicity [40]. 

ETs have received increased attention on this matter due to their chemopreventive and chemotherapeutic activities [41]. These effects seem to be induced by their high antioxidant capacity. The latter can vary according to the degree of tannin hydroxylation and it depends on iron chelation activity and on capacity of the radical scavenging [42]. 

ETs have also demonstrated the ability to inhibit angiogenesis in both in vitro and in vivo prostate cancer models. They exert this action by binding directly to VEGF receptors and by indirectly reducing endothelial growth [43].

## 5. The Effects of Tannins in Internal Medicine

HTs have shown to have multiple therapeutic applications. In fact, as discussed above, these molecules are able to exert various beneficial effects both in humans [35,44,45,46,47] and in animals [9,28,34,37,48,49]. These effects include antioxidant, antimicrobial, hypolipidemic, cardioprotective, and antineoplastic ones [7,46,50].

### 5.1. Antioxidant Effects

The accumulation of ROS can be detrimental to cells. Natural antioxidants may be a solution to avoid the occurrence of oxidative processes. Table 1 shows some studies in which the antioxidant functions of HTs deriving from chestnuts have been demonstrated. In particular, the antioxidant activity of HTs seems to reside in GA and phenolic acid, which is usually bound to glucose in gallotannins.

Dinis et al. [8] evaluated the antioxidant capacities of chestnut extracts through several biochemical assays, such as 2,2-diphenyl-1-picrylhydrazyl9 (DPPH) radical scavenging activity and 2,20-azinobis-(3-ethylbenzothiazoline-6-sulphonic acid) (ABTS). An important aspect that was taken into consideration in this study was the edaphoclimatic conditions of the chestnut cultivar, since different varieties were tested for their antioxidant capacities. It was revealed that those varieties cultivated in the coldest climatic conditions reported a higher amount of phenolic and antioxidant compounds, suggesting a higher antioxidant capacity compared to those extracts deriving from the chestnuts coming from hotter environments. Malondialdehyde (MDA), a common biomarker of oxidative stress, was measured in the plasma of broilers receiving a diet supplemented with chestnuts. The group of broilers receiving a dietary supplementation of chestnut extracts showed a significant decrease in plasma MDA, demonstrating the antioxidant nature of these compounds [9]. 

Liu et al. analyzed different plasma values which were clearly connected to the oxidative status of dairy cows [28]. The authors showed how the addition of CTs in the cows’ diet not only inhibited the lipid peroxidation, but also increased the activity of various antioxidant enzymes in their plasma and liver [28]. Moreover, no modification has been observed for what concerns the body weight of cows, the milk yield, or its composition in terms of proteins, fats, and lactose [28]. The activity of the MDA enzyme decreased after a diet supplemented with tannins, showing an improvement in terms of the general oxidative status of the dairy cows [28]. 

GA can be considered a powerful natural antioxidant able to counteract different types of ROS, such as H_2_O_2_, hydroxyl radicals, and superoxide anion. 

Samuel et al. studied how that the addition of dietary GA showed its effects after a certain period of administration and at a certain dosage. In particular, it was observed that, in the initial phase of the life of the chicks, nonsignificant effects were recorded. Despite this, encouraging results were highlighted during the growing phase, when the MDA plasma levels started to decrease [11]. In fact, higher dosages of GA were related to a linear increase in the plasma total antioxidant capacity and in the SOD activity [11].

Silva et al. [30] evaluated how multiple byproducts of a phenolic structure deriving from chestnut shells, inner shells, and leaves were able to induce antioxidant effects. The latter were ascribable to the H-atom donation, to chelation reactions, and to the inhibition of several enzymes, including topoisomerase-like ones.

These studies clearly emphasize the antioxidant capacities of CTs. In summary, they are able to reduce the lipid peroxidation, to scavenge free radicals, and to act as metal chelators in order to decrease OS.

### 5.2. Antimicrobial Effects

The increasing interest for research on the antibacterial activity of phenolic compounds is related to the rising resistance of several bacterial strains to multiple drugs [30]. Nowadays, the phenomenon of bacterial resistance is growing due to the misuse of antibiotics, and it is one of the major global concerns [51,52,53]. Table 2 summarizes a variety of studies in which tannins have shown their bactericidal activities.

Currently, the literature is scarce concerning specific studies aimed at analyzing the antimicrobial properties of phenolic compounds, which include HTs. However, due to the actual interest in alternatives to antibiotic therapy, researchers are starting to evaluate and look for natural antioxidants, which are also fundamental in the food industry. In addition, it has been demonstrated that the interaction of phenolic compounds with host-gut microbiota induces a positive impact on the microbiota composition itself [31].

ETs, found within the leaves and the bur of chestnuts, have proven to exert antimicrobial activity against several different bacterial strains [54]. 

Silva et al. [30] tested eight multidrug-resistant bacterial strains for their ET antimicrobial susceptibility. Four of them were Gram-positive bacteria, and the other four were Gram-negative. The latter included *Salmonella enteritidis*, *Escherichia coli*, *Klebsiella pneumoniae*, and *Pseudomonas aeruginosa*. Differently, Gram-positive strains included *Staphylococcus aureus*, *Staphylococcus epidermidis*, *Enterococcus faecium*, and *Enterococcus faecalis*. In addition, two Gram-positive foodborne strains were tested, such as *Bacillus cereus* and *Listeria monocytogenes* [30]. The results pointed out how the growth was inhibited in six out of ten bacterial strains. Among the Gram-positive ones, antibacterial effects were observed in all the *Staphylococcus* and *Enterococcus* strains tested. Growth inhibition was also observed within the cultures containing *Klebsiella pneumoniae* and *Pseudomonas aeruginosa* [30].

Additionally, the effects of the tannins on the growth of *E. coli* in vitro were assessed in another study. In this study, both the actions of the chestnuts and mimosa tannins were tested. The growth of the bacterial strains was reduced in a dose-dependent manner when both types of tannins were added to the cultures [49]. The important difference to underline is that the CTs were able to exert effects on all the bacterial strains tested, whilst the mimosa tannins only exerted effects on one single strain [49]. We have to take into account that, in humans, it is possible that CTs can be degraded in an aerobic environment, like the one in the intestinal tract, where they could lose their antimicrobial activity [55,56].

In this regard, the study conducted by Noce et al. [47] was aimed at treating recurrent urinary infections with the addition of an oral food supplement (OFS) as an adjuvant tool apart from the standard therapeutic approaches. Urinary tract infections (UTIs) can be considered as one of the most frequent nephron–urological conditions and, for this reason, they represent one of the main bacterial infections found [57]. This type of infection occurs more often in the female gender. This assertion was confirmed by a recent study which underlined that women have a 30-times higher risk of developing UTIs than men [58]. UTIs can also be present in patients with a kidney transplant [59] and in the cases of a familial history of UTIs, where genetics plays a role in the occurrence of the infection between first-degree relatives [60]. Noce et al. evaluated the properties of tannins and anthocyanins contained within sweet chestnut and cranberry extracts on a population affected by chronic kidney disease (CKD), and, specifically, on the onset of recurrent UTIs. Twenty-six patients were selected for the study. Out of the total patients, the OFS was administered to sixteen of them, leaving ten patients as the control group. To avoid any gender disparities, the sixteen patients receiving the OFS were divided equally into males and females [47]. The patients under the OFS treatment were asked to take one capsule per day for the six-week duration. During this period, blood and urine samples were analyzed to evaluate the possible beneficial effects. Regarding the OFS analytic composition, one capsule contained 500 mg of powder, including 6.21 mg of polyphenols, in which 4.57 mg were represented by HTs, 0.94 mg by anthocyanosides, 0.51 mg by proanthocyanidis, and, lastly, 0.18 mg by quercetin derivatives [47]. Among the female patients recruited, only five completed the pilot study, since three patients reported gastrointestinal side effects and dropped out of the study [47]. The erythrocyte sedimentation rate, a common inflammation marker, showed a significant reduction in the male OFS group, with a promising result. Despite this, the same effect was not observed in the female patients. However, improvements in the values of both the male and female urine samples were observed; namely, a reduction in leukocytes in the urinary sediment of the OFS group patients [47]. Another result, observed only in the male patients, was the reduction in urinary bacterial flora. The authors concluded that the HTs and polyphenols obtained from chestnuts seemed to exert an antimicrobial action in a gender-dependent manner. Nevertheless, it can be deducted that a daily intake of food supplements containing the previously cited extracts for a prolonged period is able to exert antimicrobial actions. However, a gender difference may be a variable to be considered [47]. In fact, other bioactive molecules, including polyphenols, have also reported different data regarding the bioavailability, pharmacodynamics, and pharmacokinetics according to gender differences. The factors that cause these differences are renal excretion, the gap between the expression of specific targets, and metabolic variations, such as the gender-related differences of cytochrome P450 mono-oxygenate enzyme activity [61,62,63].

Another study aimed at evaluating the possible antimicrobial effects of tannins was conducted in vitro by Anderson et al. [38]. In particular, these authors tested the bactericidal effects of a combination of both hydrolyzable and condensed tannins on *Campylobacter jejuni*, showing interesting results. 

Mannelli et al. [64] analyzed the effects of tannins deriving from chestnuts as feeding supplements and antimicrobial compounds in the meat quality of broilers. The results concluded that a mixture of these supplements represented a valuable alternative to antibiotics. Similar effects were observed in the study conducted by Elizondo et al. [65], which demonstrated how a combination of quebracho and CTs was able to inhibit the in vitro growth of *Clostridium perfringens*.

Mastitis is a global disease among cows [66]. It may cause a reduced supply of milk and, consequently, economic damage [18]. Different antibiotics, such as gentamicin and penicillin, have been used to treat this pathological condition. However, the antibiotic treatment not always successful. HTs are used as antimicrobial agents due to their properties, which include the inhibition of extracellular microbial enzymes and the deprivation of substrates and essential minerals (like zinc and iron) to the microbe [67]. In vitro testing revealed that penicillin had the highest antibacterial activity against bacterial strains, such as *Staphylococcus aureus*, *Streptococcus uberis*, and *Pseudomonas aeruginosa*. However, it was not able to inhibit the growth of *Escherichia coli* and *Klebsiella pneumoniae* [68]. In order to overcome this limit, Prapaiwong et al. [68] analyzed the in vitro effects of HT extracts coming from *Castanea sativa* upon specific species of dairy cows suffering from subclinical mastitis. The authors highlighted the synergistic effects of specific antibiotics (such as gentamicin) and chestnut extracts on the inhibition of bacteria growth [68]. 

### 5.3. Effects on the Lipid Metabolism

The prevalence of obesity and lipid metabolism disorders is growing worldwide [19]. Back in 2015, the Global Burden of Disease (GBD) reported that 1.77 million children and 6.37 million adults around the world were obese. Moreover, it was stated that the number of health problems caused by obesity doubled compared to the 1980s [69]. Generally, adipose tissue is present in specific body areas, such as around the internal organs, in the breast tissue, in the abdominal area, or more scattered in the subcutaneous tissue. Fat tissue also has some positive effects, including the capacity to act as an insulator against the heat and cold, and mainly in maintaining homeostasis and energy balance [70]. Nevertheless, it is important to underline that an abundant accumulation of adipose tissue can lead to obesity. A person develops obesity if there is an imbalance between the energy intake and the energy expenditure, which will lead to the growth of adipocytes. This process is considered pathological and it is regulated by specific transcription factors that have an impact on adipocyte differentiation [71]. Concerning the lipid metabolism and obesity, tannins seem to play a pivotal role (Table 3).

**Figure 6:**
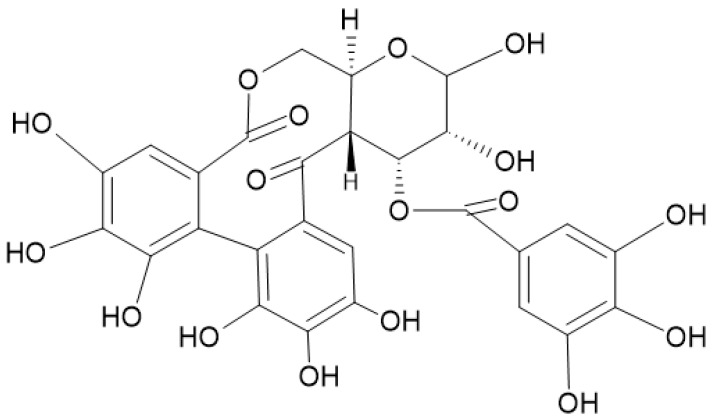
O-galloyl-4,6-(s)-HHDP-β-D-glucopyranose is a metabolite of tannins which has shown to induce promising antineoplastic actions.

Yasuda et al. [19] observed how, by increasing the concentrations of chestnut polyphenols administered to mice, a lesser accumulation of fat percentages was noticed, suggesting that the higher the concentration of polyphenols, the higher the inhibition of fat accumulation. It is interesting to observe how a concentration of 12.5 μg/mL of chestnut polyphenols exhibited the same effect on a solution containing 25 μg/mL of EGCG (Figure 6), demonstrating how chestnut polyphenols have a stronger capacity in inhibiting fat accumulation compared to a source of green tea polyphenols [19].

Noh et al. [72] attempted to utilize molecules deriving from natural sources (such as chestnut extract) to assess the effects on the lipid metabolism, more specifically on an induced condition of hepatic steatosis in mice. In order to evaluate the effects of the chestnut extract on the disease development and progression, evaluations, such as the measurement of the hepatic lipid accumulation, the mRNA expression of the lipogenic genes, and the activity of those enzymes involved in the hepatic lipid metabolism, were made [72]. The results reported that the hepatic concentrations of triacylglycerol diminished by 69.7% compared to the control group and the total cholesterol also decreased by 37.5%. Apart from these encouraging results, several genes showed a reduction in terms of the mRNA expression, revealing their role in the inhibition mechanisms responsible for hepatic steatosis. These genes are:

- Sterol regulatory element binding protein 1-c;
- HMG-CoA reductase;
- Fatty acid synthase (FAS);
- Acetyl CoA acyltransferase;
- Acetyl CoA carboxylase.

Among these genes, the hepatic FAS and HMG-CoA reductase activities were significantly reduced in the group who received the chestnut extracts, showing that these enzymes mediate and play crucial roles in the lipid biogenesis [72]. This study demonstrated that these enzymes have an impact on lipolysis and on the lipid metabolism [72]. 

Youn et al. [73] analyzed if the byproducts deriving from chestnuts were able to exert beneficial effects on the lipid metabolism. Antiadipogenic and antioxidant properties were shown by these byproducts, suggesting their utility as beneficial molecules. In the study, it was shown how a concentration of 100 μg/mL inhibited the adipogenesis of 3T3-L1 cells in mice by 96.5% [73]. 

### 5.4. Cardioprotective Effects

The main causes of morbidity and mortality around the world are cardiovascular diseases [74]. Chronic kidney disease, obesity, and diabetes mellitus may contribute to their onset. Moreover, OS plays an important role in the etiology of cardiovascular pathologies, since it may induce DNA damage both in the nucleus and in the mitochondria. The latter are an important source of ROS production in different organs, such as the heart, which has a high metabolic activity [75]. OS-mediated injury may lead to apoptosis or necrosis of the cardiac tissue [76]. The polyphenols’ antioxidant actions exert beneficial effects to heart health. Polyphenols, rather than exerting direct antioxidant effects, are able to interact with specific cellular signaling pathways (such as the MAP kinase, ERK, p38, and PI3 kinase/Akt signaling cascade), and through these, they are able to exert an indirect antioxidant effect in both physiological and pathological conditions. Some of these pathways influence NO production, an important vasodilator, that impact blood pressure and cardiovascular diseases [77]. Several studies have emphasized the significance of HTs as cardioprotective elements (Table 4).

ETs can exert multiple cardioprotective effects, namely, antithrombotic, antiatherogenic, antiangiogenic, and anti-inflammatory ones [44,46]. 

Foods rich in polyphenols, anthocyanins, and isoflavones may improve blood pressure levels. Additionally, it is important to specify that no single food is able to directly counteract the occurrence of arterial hypertension. However, a plant-based diet could have a positive impact on the mechanisms which regulate arterial blood pressure [78,79,80]. These mechanisms, by which polyphenols affect the endothelial functions and reduce the risk of arterial hypertension, include the increase in vascular smooth muscle relaxation, the increase in the synthesis of antioxidant cell defenses through regulation of specific targets, including the MAPK, JAK/STAT, and nuclear factor-κB (NF-κB) signaling pathways, the increase in mitochondrial functions, the increase in the secretion of NO, the decrease in the oxidized-LDL concentration, and the decrease in TNF-α, IL-1, and IL-6 [7].

Another in vitro study demonstrated that tannic acid is able to induce the transcriptor factor Kruppel-like-factor 2 (KLF2) expression via the ERK5/MEF2 pathway. KLF2 plays a pivotal role in the vascular endothelium, inducing anti-inflammatory and antiatherogenic effects [81].

### 5.5. Antineoplastic Effects

Studies have indicated that those populations consuming a high quantity of plant-derived foods have low occurrence rates of cancer. Antineoplastic properties have been attributed to polyphenols, such as the capacity to interact with different cancerous development stages, including initiation processes, progression phases, and spread [82]. Phenolic molecules are able to modulate these mechanisms by regulating cell signaling cascades. In detail, transcription factors and various kinase enzymes regulate the expression of those genes involved in apoptosis, in cell survival, or in cell-cycle arrest. In addition, polyphenols are also able to regulate the interaction between the growth factors and target receptors involved in specific signaling effects [82]. Table 5 presents a list of studies which highlight the antineoplastic effects induced by different polyphenolic molecules deriving from plant-based food.

The pleiotropic effects of phytochemicals deriving from edible fruits and vegetables are recognized as a possibility to reduce cancer incidence and mortality [42]. The synergism between cytoprotective and cytotoxic effects occurs against normal and neoplastic cells, respectively, representing a great advantage of polyphenols as anticancer agents [83]. 

ETs have shown to act against numerous types of cancers. This variety of functions is attributable to the pleiotropism of these molecules. Examples include the antiproliferative effects in liver, lung, colon, and prostate cancers [45]. Indeed, ETs have received an increased attention on their anticancerous properties due to the chemopreventive and chemotherapeutic activities [41]. Together with other polyphenols, ETs could also be used to increase the susceptibility of chemotherapeutic drugs. This new adjuvant therapy could empower the chemotherapeutic efficacy, decreasing the chemotherapeutic drugs dosage. In this way, the occurrence of side effects, which often represent a limit for continuing the chemotherapy, can be reduced [45]. 

Sorice et al. [84] analyzed the modulation of cancerous cell growth under the effects of polyphenolic compound extracts. The study consisted in testing six different human cell lines to evaluate the potential anticancer effects of chestnut extracts. HepG2 cells are commonly used as a human cell line for liver carcinoma. Promising results demonstrated that, after a 48 h treatment with 137 μg/mL of a solution containing the chestnut extract, the HepG2 cells showed an apoptosis increase of 52.21% and also a modulatory action on the cell cycle progression [84]. Tumor cells are dependent on glucose, which is one of their most important nutrients. It is well known that neoplastic cells consume more glucose than physiological ones through a glycolysis pathway which converts pyruvate into lactate. The deprivation of this fundamental nutrient may lead to cell death or necrosis. In a study by Sorice et al., the decrease in the glucose levels in human HepG2 cells was prominent [84]. 

Woo et al. [85] examined 89 extracts of different edible plants to evaluate their inhibitory action against the NF-κB pathway, which is actively involved in the drug-resistance mechanism of cancer stem cells (CSCs). The authors highlighted how the leaf extract derived from *Castanea sativa* significantly reduced the nuclear translocation of Nrf2, suggesting that supplementation with chestnut extracts would appear to be useful in order to increase the susceptibility of breast CSCs to antitumor drugs.

**Table 5:** Antineoplastic effects of plant polyphenolics including tannins.

Type of Study	Pathways	Beneficial Effects	Citation
Human study	Carcinogenesis and tumor growth PI3K	↓ Akt/NF-κB ↑ Apoptosis Cell-cycle arrest	[45]
Human study	Apoptosis, cell-cycle arrest ↓ Invasion and metastasis	Problem arises with administration to humans because of low bioavailability; however, novel drug-delivery systems may be a solution	[86]
In vitro study	↓ Nrf2	The suppression of Nrf2 increases chemosensitivity to Paclitaxel in BCSC	[85]
In vitro and animal study	↓ NF-κB and AP-1	↓ Angiogenesis and metastasis capacity	[82]
In vitro study	↑ Hepatoblastoma HepG2 cell apoptosis ↓ TNF-α, VEGF	Beneficial effects of tannins on cell proliferation and apoptosis of tumor cells	[84]

Abbreviations: 2PI3K, phosphoinositide 3-kinase; Akt, protein kinase-B; AP-1, activator protein 1; BCSCs, breast cancer stem cells; NF-κB, nuclear factor kappa-light-chain-enhancer of activated B cells; Nrf2, NF-E2-related factor; TNF, tumor necrosis factor; VEGF, vascular endothelial growth factor; ↓ decrease; ↑ increase.

Proinflammatory pathways, such as NF-κB signaling, lead to inflammation, the activation of the immunological response, and the transcription of genes involved in cell survival, namely, Bcl-x. A type of cancer characterized by chronic inflammation is prostate cancer. Apart from the direct action towards tumor cells, the cytostatic/cytotoxic activities of ETs might suggest an additional mechanism by which these compounds exert a chemopreventive potential by avoiding neoplastic cells from replicating and turning into more malignant stages [87]. 

A study pointed out the antiproliferative activity shown by 1,3-di-O-galloyl-4,6-(s)-HHDP-β-D-glucopyranose (Figure 6) in human Hep-G2 liver cancerous cells because of an altered regulation of 25 microRNA genes, including the let-7 family: miR-526b, miR-373, and miR-370. These genes were identified as potential targets against cell differentiation and proliferation [88]. 

## 6. Conclusions

In conclusion, several studies have highlighted the potential beneficial effects of HTs deriving from chestnuts. In fact, they have the capacity to exert antioxidant effects through different pathways. In addition to the antioxidant functions, some studies have revealed how these molecules can induce antimicrobial, antineoplastic, hypolipemic, and cardioprotective actions. The latter also seems to be related to the improvement of endothelial dysfunction. 

Furthermore, HTs showed encouraging results in the synergism with chemotherapeutic drugs. In fact, the concurrent administration of HTs with some chemotherapeutic drugs may provide substantial advances in the treatment of some types of cancer.

Therefore, HTs obtained by a circular economy model can represent a new adjuvant therapy, either if assumed as an oral food supplement or as a functional food.

## Figures and Tables

**Table 1:** Antioxidant effects of chestnut hydrolysable tannins.

Type of Study	Pathways	Beneficial Effects	Citation
Animal study	SOD, GSH-Px, T-AOC, MDA	↓ BW, plasma MDA, lipid peroxidation ↑ antioxidant enzymes	[28]
Animal study	MDA, T-AOC, SOD	↓ MDA ↑ T-AOC, SOD, growth and health benefits	[11]
In vitro study	Donation of H atom, inhibition of topoisomerase-like enzymes, chelation reactions	↑ Antioxidant activity	[30]

Abbreviations: BW, body weight; GSH-Px, glutathione peroxidase; MDA, malondialdehyde; SOD, superoxide dismutase; T-AOC, total antioxidant capacity; ↓ decrease; ↑ increase.

**Table 2:** Antimicrobial effects of chestnut hydrolysable tannins.

Type of Study	Pathways	Beneficial Effects	Citation
In vitro study	Effectiveness against Gram-positive and Gram-negative bacterial strains	*Staphylococcus epidermidis* was susceptible to all CEs	[30]
Human study	↓ ESR	↓ Leukocytes in urine, urinary bacterial flora, UTIs	[47]
In vitro and in vivo study	Bacterial growth inhibition	Bactericidal effects towards *E. coli*	[49]

Abbreviations: CE, chestnut extract; ESR, erythrocyte sedimentation rate; UTIs, urinary tract infections; ↓ decrease.

**Table 3:** Effects on the lipid metabolism of chestnut hydrolyzable tannins.

Type of Study	Pathways	Beneficial Effects	Citation
In vitro, animal, and human study	Lipase inhibitory acitivity Fat accumulation TG, FFA	↓ Adiposity, TG, FFA, visceral fat area	[19]
Animal study	↓ Hepatic TAG, plasma lipid levels	Suppression of lipid synthesis ↑ FAOX	[72]
In vitro study	↓ ROS, 3T3-L1 adipogenesis,	Suppression of lipid synthesis Antioxidant activity	[73]

Abbreviations: FAOX, fatty acid oxidation; FFA, free fatty acids; ROS, reactive oxygen species; TAG, triacylglycerol; TG, triglycerides; ↓ decrease; ↑ increase.

**Table 4:** Cardioprotective effects of chestnut hydrolyzable tannins.

Type of Study	Pathways	Beneficial Effects	Citation
Animal study	ROS formation, cholinergic and adrenergic responses	↓ ROS ↓ Noradrenaline contraction Transient negative chronotropic effects Positive ionotropic effects	[50]
In vitro and in vivo study (both animal and human)	Free radical-scavenging activity	In vitro testing shows positive effects on vascular health Due to bioavailability reasons, in vivo testing provides deficient results	[46]

Abbreviations: ROS, reactive oxygen species; ↓ decrease.

## Data Availability

Not applicable.
